# Plasma phosphorylated-tau181 as a predictive biomarker for Alzheimer’s amyloid, tau and FDG PET status

**DOI:** 10.1038/s41398-021-01709-9

**Published:** 2021-11-13

**Authors:** Xue-Ning Shen, Yu-Yuan Huang, Shi-Dong Chen, Yu Guo, Lan Tan, Qiang Dong, Jin-Tai Yu, Alzheimer’s Disease Neuroimaging Initiative, Michael W. Weiner, Paul Aisen, Ronald Petersen, Clifford R. Jack, William Jagust, John Q. Trojanowki, Arthur W. Toga, Laurel Beckett, Robert C. Green, Andrew J. Saykin, John C. Morris, Richard J. Perrin, Leslie M. Shaw, Maria Carrillo, William Potter, Lisa Barnes, Marie Bernard, Hector González, Carole Ho, John K. Hsiao, Jonathan Jackson, Eliezer Masliah, Donna Masterman, Ozioma Okonkwo, Richard Perrin, Laurie Ryan, Nina Silverberg, Adam Fleisher, Juliet Fockler, Cat Conti, Dallas Veitch, John Neuhaus, Chengshi Jin, Rachel Nosheny, Miriam Ashford, Derek Flenniken, Adrienne Kormos, Gustavo Jimenez, Michael Donohue, Devon Gessert, Jennifer Salazar, Caileigh Zimmerman, Yuliana Cabrera, Sarah Walter, Garrett Miller, Godfrey Coker, Taylor Clanton, Lindsey Hergesheimer, Stephanie Smith, Olusegun Adegoke, Payam Mahboubi, Shelley Moore, Jeremy Pizzola, Elizabeth Shaffer, Brittany Sloan, Danielle Harvey, Bret Borowski, Chad Ward, Christopher Schwarz, David Jones, Jeff Gunter, Kejal Kantarci, Matthew Senjem, Prashanthi Vemuri, Robert Reid, Nick C. Fox, Ian Malone, Paul Thompson, Sophia I. Thomopoulos, Talia M. Nir, Neda Jahanshad, Charles DeCarli, Alexander Knaack, Evan Fletcher, Duygu Tosun-Turgut, Stephanie Rossi Chen, Mark Choe, Karen Crawford, Paul A. Yushkevich, Sandhitsu Das, Robert A. Koeppe, Eric M. Reiman, Kewei Chen, Chet Mathis, Susan Landau, Richard Perrin, Nigel J. Cairns, Erin Householder, Erin Franklin, Haley Bernhardt, Lisa Taylor-Reinwald, Leslie M. Shaw, John Q. Trojanowki, Magdalena Korecka, Michal Figurski, Karen Crawford, Scott Neu, Andrew J. Saykin, Kwangsik Nho, Shannon L. Risacher, Liana G. Apostolova, Li Shen, Tatiana M. Foroud, Kelly Nudelman, Kelley Faber, Kristi Wilmes, Leon Thal, Zaven Khachaturian, John K. Hsiao, Lisa C. Silbert, Betty Lind, Rachel Crissey, Jeffrey A. Kaye, Raina Carter, Sara Dolen, Joseph Quinn, Lon S. Schneider, Sonia Pawluczyk, Mauricio Becerra, Liberty Teodoro, Karen Dagerman, Bryan M. Spann, James Brewer, Helen Vanderswag, Adam Fleisher, Jaimie Ziolkowski, Judith L. Heidebrink, Lisa Zbizek-Nulph, Joanne L. Lord, Colleen S. Albers, David Knopman, Kris Johnson, Javier Villanueva-Meyer, Valory Pavlik, Nathaniel Pacini, Ashley Lamb, Joseph S. Kass, Rachelle S. Doody, Victoria Shibley, Munir Chowdhury, Susan Rountree, Mimi Dang, Yaakov Stern, Lawrence S. Honig, Akiva Mintz, Beau Ances, David Winkfield, Maria Carroll, Georgia Stobbs-Cucchi, Angela Oliver, Mary L. Creech, Mark A. Mintun, Stacy Schneider, David Geldmacher, Marissa Natelson Love, Randall Griffith, David Clark, John Brockington, Daniel Marson, Hillel Grossman, Martin A. Goldstein, Jonathan Greenberg, Effie Mitsis, Raj C. Shah, Melissa Lamar, Patricia Samuels, Ranjan Duara, Maria T. Greig-Custo, Rosemarie Rodriguez, Marilyn Albert, Chiadi Onyike, Leonie Farrington, Scott Rudow, Rottislav Brichko, Stephanie Kielb, Amanda Smith, Balebail Ashok Raj, Kristin Fargher, Martin Sadowski, Thomas Wisniewski, Melanie Shulman, Arline Faustin, Julia Rao, Karen M. Castro, Anaztasia Ulysse, Shannon Chen, Mohammed O. Sheikh, Jamika Singleton-Garvin, P. Murali Doraiswamy, Jeffrey R. Petrella, Olga James, Terence Z. Wong, Salvador Borges-Neto, Jason H. Karlawish, David A. Wolk, Sanjeev Vaishnavi, Christopher M. Clark, Steven E. Arnold, Charles D. Smith, Gregory A. Jicha, Riham El Khouli, Flavius D. Raslau, Oscar L. Lopez, MaryAnn Oakley, Donna M. Simpson, Anton P. Porsteinsson, Kim Martin, Nancy Kowalski, Melanie Keltz, Bonnie S. Goldstein, Kelly M. Makino, M. Saleem Ismail, Connie Brand, Gaby Thai, Aimee Pierce, Beatriz Yanez, Elizabeth Sosa, Megan Witbracht, Brendan Kelley, Trung Nguyen, Kyle Womack, Dana Mathews, Mary Quiceno, Allan I. Levey, James J. Lah, Ihab Hajjar, Janet S. Cellar, Jeffrey M. Burns, Russell H. Swerdlow, William M. Brooks, Daniel H. S. Silverman, Sarah Kremen, Liana Apostolova, Kathleen Tingus, Po H. Lu, George Bartzokis, Ellen Woo, Edmond Teng, Neill R. Graff-Radford, Francine Parfitt, Kim Poki-Walker, Martin R. Farlow, Ann Marie Hake, Brandy R. Matthews, Jared R. Brosch, Scott Herring, Christopher H. van Dyck, Adam P. Mecca, Susan P. Good, Martha G. MacAvoy, Richard E. Carson, Pradeep Varma, Howard Chertkow, Susan Vaitekunis, Chris Hosein, Sandra Black, Bojana Stefanovic, Chris (Chinthaka) Heyn, Ging-Yuek Robin Hsiung, Ellen Kim, Benita Mudge, Vesna Sossi, Howard Feldman, Michele Assaly, Elizabeth Finger, Stephen Pasternak, Irina Rachinsky, Andrew Kertesz, Dick Drost, John Rogers, Ian Grant, Brittanie Muse, Emily Rogalski, Jordan Robson, M.-Marsel Mesulam, Diana Kerwin, Chuang-Kuo Wu, Nancy Johnson, Kristine Lipowski, Sandra Weintraub, Borna Bonakdarpour, Nunzio Pomara, Raymundo Hernando, Antero Sarrael, Howard J. Rosen, Bruce L. Miller, David Perry, Raymond Scott Turner, Kathleen Johnson, Brigid Reynolds, Kelly MCCann, Jessica Poe, Reisa A. Sperling, Keith A. Johnson, Gad A. Marshall, Christine M. Belden, Alireza Atri, Bryan M. Spann, Kelly A. Clark, Edward Zamrini, Marwan Sabbagh, Ronald Killiany, Robert Stern, Jesse Mez, Neil Kowall, Andrew E. Budson, Thomas O. Obisesan, Oyonumo E. Ntekim, Saba Wolday, Javed I. Khan, Evaristus Nwulia, Sheeba Nadarajah, Alan Lerner, Paula Ogrocki, Curtis Tatsuoka, Parianne Fatica, Evan Fletcher, Pauline Maillard, John Olichney, Charles DeCarli, Owen Carmichael, Vernice Bates, Horacio Capote, Michelle Rainka, Michael Borrie, T-Y Lee, Rob Bartha, Sterling Johnson, Sanjay Asthana, Cynthia M. Carlsson, Allison Perrin, Anna Burke, Douglas W. Scharre, Maria Kataki, Rawan Tarawneh, Brendan Kelley, David Hart, Earl A. Zimmerman, Dzintra Celmins, Delwyn D. Miller, Laura L. Boles Ponto, Karen Ekstam Smith, Hristina Koleva, Hyungsub Shim, Ki Won Nam, Susan K. Schultz, Jeff D. Williamson, Suzanne Craft, Jo Cleveland, Mia Yang, Kaycee M. Sink, Brian R. Ott, Jonathan Drake, Geoffrey Tremont, Lori A. Daiello, Jonathan D. Drake, Marwan Sabbagh, Aaron Ritter, Charles Bernick, Donna Munic, Akiva Mintz, Abigail O’Connelll, Jacobo Mintzer, Arthur Wiliams, Joseph Masdeu, Jiong Shi, Angelica Garcia, Marwan Sabbagh, Paul Newhouse, Steven Potkin, Stephen Salloway, Paul Malloy, Stephen Correia, Smita Kittur, Godfrey D. Pearlson, Karen Blank, Karen Anderson, Laura A. Flashman, Marc Seltzer, Mary L. Hynes, Robert B. Santulli, Norman Relkin, Gloria Chiang, Michael Lin, Lisa Ravdin, Athena Lee, Ron Petersen, Thomas Neylan, Jordan Grafman, Tom Montine, Sarah Danowski, Catherine Nguyen-Barrera, Shannon Finley, Danielle Harvey, Michael Donohue, Matthew Bernstein, Norm Foster, Tatiana M. Foroud, Steven Potkin, Li Shen, Kelley Faber, Sungeun Kim, Kwangsik Nho, Kristi Wilmes, Helen Vanderswag, Adam Fleisher, Ajay Sood, Kimberly S. Blanchard, Debra Fleischman, Maria T. Greig, Bonnie Goldstein, Kimberly S. Martin, Gaby Thai, Aimee Pierce, Christopher Reist, Beatriz Yanez, Elizabeth Sosa, Megan Witbracht, Carl Sadowsky, Walter Martinez, Teresa Villena, Howard Rosen, Elaine R. Peskind, Eric C. Petrie, Gail Li, Jerome Yesavage, Joy L. Taylor, Steven Chao, Jaila Coleman, Jessica D. White, Barton Lane, Allyson Rosen, Jared Tinklenberg, Gustavo Jimenez-Maggiora, Erin Drake, Mike Donohue, Craig Nelson, David Bickford, Meryl Butters, Michelle Zmuda, Bret Borowski, Jeff Gunter, Matt Senjem, Kejal Kantarci, Chad Ward, Denise Reyes, Kelley M. Faber, Kelly N. Nudelman, Yiu Ho Au, Kelly Scherer, Daniel Catalinotto, Samuel Stark, Elise Ong, Dariella Fernandez, Michelle Zmuda

**Affiliations:** 1 Department of Neurology and Institute of Neurology, Huashan Hospital, Shanghai Medical College, Fudan University, Shanghai, China; 2 Department of Neurology, Qingdao Municipal Hospital, Qingdao University, Qingdao, China; 3 University of California, San Francisco, San Francisco, USA; 4 University of Southern California, Los Angeles, USA; 5 Mayo Clinic, Rochester, Rochester, USA; 6 University of California, Berkeley, Berkeley, USA; 7 University of Pennsylvania, Philadelphia, USA; 8 University of California, Davis, Davis, USA; 9 BWH/HMS, Boston, USA; 10 Indiana University, Bloomington, USA; 11 Washington University St. Louis, St. Louis, USA; 12 Alzheimer’s Association, Chicago, USA; 13 National Institute of Mental Health, Rochville, USA; 14 Rush University, Chicago, USA; 15 NIA, Bethesda, USA; 16 University of California, San Diego, San Diego, USA; 17 Denali Therapeutics, South San Francisco, USA; 18 NIH, Bethesda, USA; 19 Massachusetts General Hospital, Boston, USA; 20 Biogen, Cambridge, USA; 21 University of Wisconsin, Madison, Madison, USA; 22 Washington University, St. Louis, USA; 23 Eli Lilly, Indianapolis, USA; 24 NCIRE/The Vererans Health Research Institute, San Francisco, USA; 25 Mayo Clinic, Scottsdale, USA; 26 University College London, London, UK; 27 University of Southern California School of Medicine, Los Angeles, USA; 28 University of Michigan, Ann Arbor, USA; 29 Banner Alzheimer’s Institute, Phoenix, USA; 30 University of Pittsburgh, Pittsburgh, USA; 31 Perelman School of Medicine, University of Pennsylvania, Philadelphia, USA; 32 Indiana University School of Medicine, Indianapolis, USA; 33 UPenn School of Medicine, Philadelphia, USA; 34 NCRAD/Indiana University School of Medicine, Indianapolis, USA; 35 Prevent Alzheimer’s Disease, 2020 Rockville, USA; 36 National Institute on Aging, Bethesda, USA; 37 Oregon Health & Science University, Portland, USA; 38 University of California – San Diego, San Diego, USA; 39 Baylor College of Medicine, Houston, USA; 40 Columbia University Medical Center, New York, USA; 41 Washington University, St. Louis, St. Louis, USA; 42 University of Alabama - Birmingham, Birmingham, USA; 43 Mount Sinai School of Medicine, New York, USA; 44 Rush University Medical Center, Chicago, USA; 45 Wien Center, Miami Beach, USA; 46 Johns Hopkins University, Baltimore, USA; 47 University of South Florida: USF Health Byrd Alzheimer’s Institute, Tampa, USA; 48 New York University, New York, USA; 49 Duke University Medical Center, Durham, USA; 50 University of Kentucky, Lexington, USA; 51 University of Rochester Medical Center, New York, USA; 52 University of California Irvine IMIND, Irvine, USA; 53 University of Texas Southwestern Medical School, Dallas, USA; 54 Emory University, Atlanta, USA; 55 University of Kansas, Medical Center, Kansas, USA; 56 University of California, Los Angeles, Los Angeles, USA; 57 Mayo Clinic, Jacksonville, Jacksonville, USA; 58 Yale University School of Medicine, New Haven, USA; 59 McGill Univ., Montreal-Jewish General Hospital, Montréal, Canada; 60 Sunnybrook Health Sciences, Ontario, Toronto, Canada; 61 U.B.C. Clinic for AD & Related Disorders, Vancouver, Canada; 62 St. Joseph’s Health Care, Petaluma, USA; 63 Northwestern University, Evanston, USA; 64 Nathan Kline Institute, Orangeburg, USA; 65 Georgetown University Medical Center, Washington, USA; 66 Brigham and Women’s Hospital, Boston, USA; 67 Banner Sun Health Research Institute, Sun City, USA; 68 Boston University, Boston, USA; 69 Howard University, Washington, USA; 70 Case Western Reserve University, Cleveland, USA; 71 University of California, Davis – Sacramento, Sacramento, USA; 72 Dent Neurologic Institute, Orchard Park, USA; 73 Parkwood Institute, London, Canada; 74 University of Wisconsin, Madison, USA; 75 Ohio State University, Columbus, USA; 76 Albany Medical College, Albany, USA; 77 University of Iowa College of Medicine, Iowa City, USA; 78 Wake Forest University Health Sciences, Winston Salem, USA; 79 Rhode Island Hospital, Providence, USA; 80 Cleveland Clinic Lou Ruvo Center for Brain Health, Las Vegas, USA; 81 Roper St. Francis Healthcare, Charleston, USA; 82 Houston Methodist Neurological Institute, Houston, USA; 83 Barrow Neurological Institute, Phoenix, USA; 84 Vanderbilt University Medical Center, Nashville, USA; 85 Long Beach VA Neuropsychiatric Research Program, Long Beach, USA; 86 Butler Hospital Memory and Aging Program, Providence, USA; 87 Neurological Care of CNY, East Syracuse, USA; 88 Hartford Hospital, Olin Neuropsychiatry Research Center, Hartford, USA; 89 Dartmouth-Hitchcock Medical Center, Lebanon, USA; 90 Cornell University, Ithaca, USA; 91 Rehabilitation Institute of Chicago, Feinberg School of Medicine, Northwestern University, Chicago, USA; 92 University of Washington, Seattle, USA; 93 University of Utah, Salt Lake City, USA; 94 UC Irvine, Irvine, USA; 95 NCRAD, Indianapolis, USA; 96 University of California, Irvine, Irvine, USA; 97 Premiere Research Inst (Palm Beach Neurology), West Palm Beach, USA; 98 Stanford University, Stanford, USA; 99 BWM/HMS, Boston, USA

**Keywords:** Diagnostic markers, Predictive markers

## Abstract

Plasma phosphorylated-tau181 (p-tau181) showed the potential for Alzheimer’s diagnosis and prognosis, but its role in detecting cerebral pathologies is unclear. We aimed to evaluate whether it could serve as a marker for Alzheimer’s pathology in the brain. A total of 1189 participants with plasma p-tau181 and PET data of amyloid, tau or FDG PET were included from ADNI. Cross-sectional relationships of plasma p-tau181 with PET biomarkers were tested. Longitudinally, we further investigated whether different p-tau181 levels at baseline predicted different progression of Alzheimer’s pathological changes in the brain. We found plasma p-tau181 significantly correlated with brain amyloid (Spearman *ρ* = 0.45, *P* < 0.0001), tau (0.25, *P* = 0.0003), and FDG PET uptakes (−0.37, *P* < 0.0001), and increased along the Alzheimer’s continuum. Individually, plasma p-tau181 could detect abnormal amyloid, tau pathologies and hypometabolism in the brain, similar with or even better than clinical indicators. The diagnostic accuracy of plasma p-tau181 elevated significantly when combined with clinical information (AUC = 0.814 for amyloid PET, 0.773 for tau PET, and 0.708 for FDG PET). Relationships of plasma p-tau181 with brain pathologies were partly or entirely mediated by the corresponding CSF biomarkers. Besides, individuals with abnormal plasma p-tau181 level (>18.85 pg/ml) at baseline had a higher risk of pathological progression in brain amyloid (HR: 2.32, 95%CI 1.32–4.08) and FDG PET (3.21, 95%CI 2.06–5.01) status. Plasma p-tau181 may be a sensitive screening test for detecting brain pathologies, and serve as a predictive biomarker for Alzheimer’s pathophysiology.

## Background

Amyloid accumulation, tau deposits and neurodegeneration are the most representative pathological features of Alzheimer’s disease (AD). Detection of these pathologies are vital for screening out the target population at risk among people with intact cognition or mild cognitive impairment (MCI). Positron emission tomography (PET) and cerebrospinal fluid (CSF) are considered to be the most effective methods for detecting and tracking pathological changes in the brain. However, PET neuroimaging as a sensitive technique is hard to be added into the clinical practice due to its huge cost and radioactive burden [1]. Measurements of CSF biomarkers are also difficult to be employed in large-scale screening, because of its invasiveness and the harsh requirement for skilled operators [2]. There is a substantial need for less invasive and lower cost biomarkers in the periphery, which could be used in the clinical routine and community screening. Currently, more researchers engage in the discovery of plasma biomarkers to implement the biological definition of AD and to enrich the target population for clinical trials [3, 4].

Most studies focused on the correlations between plasma amyloid-β (Aβ) and clinical or pathologic aspects of AD [5–8], whereas a few recent researches have shifted the focus to plasma phosphorylated-tau (p-tau) [9–11]. Though peripheral measurements of the phosphorylated-tau were still difficult at present because of its low concentration, recent studies have demonstrated that detection of this marker may be promising [3]. It is found that plasma p-tau showed similar trajectory with the CSF p-tau [12]. AD patients had increased levels of p-tau in the plasma, while the combination of plasma and CSF corresponding tau protein could obviously improve diagnostic accuracy [9]. Besides, plasma p-tau and its combination with amyloid were both significantly correlated with tau deposition in the brain [13].

Plasma p-tau181 is currently under consideration to be implemented in the clinical practice, for that it was proved to elevate along with the clinical severity of AD [10]. It also tended to be a sensitive and specific predictor for brain amyloidosis and tau pathology [10, 14], differentiating AD from other neurodegenerative disorders [14]. Yet there lack either CSF/PET information or longitudinal data in previous studies, which limited the full insight into the potential role of plasma p-tau181 in the pathological process of AD. In the present study, we aimed at exploring whether the plasma p-tau181: (1) correlated with amyloid PET, tau PET and FDG PET which represented brain neurodegeneration; (2) differentiated between PET status or across clinical diagnosis in AD continuum; (3) could be used as a screening test for detecting abnormal pathological changes in the brain; (4) could predict pathological progression.

## Materials and methods

### ADNI database

The Alzheimer’s Disease Neuroimaging Initiative (ADNI) is a global and multi-centered research project that actively supports the investigation and development of treatments that slow or stop the progression of AD. Funded as a private-public partnership, ADNI began in 2004, recruiting participants who are followed and reassessed over time. In this study, researchers track the disease progression of AD dementia in the human brain, along with genetic, neuroimaging, biomarkers and clinical information. This longitudinal research was approved by institutional review boards at each site, and written informed consent was obtained on human experimentation at each institution. More detailed information could be sought at http://adni.loni.usc.edu/study-design.

### Recruitment criteria for participants

Participants from ADNI were enrolled in this study if they possessed available data as below: (1) plasma p-tau181 data, (2) amyloid, tau, or FDG PET data, (3) clinical, genetic information and neuropsychological assessments. AD patients were diagnosed as with the Mini-Mental State Examination (MMSE) score of between 20 and 26, a global Clinical Dementia Rating of 0.5 or 1 and a sum-of-boxes Clinical Dementia Rating (CDR-SB) of 1.0–9.0, conforming to the National Institute of Neurological and Communication Disorders/Alzheimer’s Disease and Related Disorders Association criteria [15]. Patients diagnosed as amnestic MCI were those with the MMSE score of between 24 to 30 and a CDR-SB score of at least 0.5. MCI patients were further dichotomized into progressive or stable MCI groups, in which the former patients conversed to AD dementia in two years from the first measurements of plasma p-tau181 whereas the latter patients remained stable during this period. Cognitively normal controls were defined as those who had the MMSE score no less than 24 and a CDR-SB score of 0 or 0.5.

### Plasma and CSF biomarker collection and measurement

Plasma p-tau181 data from ADNI was provided by the Clinical Neurochemistry Laboratory, University of Gothenburg, Sweden. Plasma p-tau181 was generated by the Single Molecule array (Simoa) technique, analyzing by an in-house assay that used the combination of two monoclonal antibodies (Tau12 and AT270) [14]. Data of CSF Aβ_1–42_, t-tau, and p-tau181 were from the Department of Pathology & Laboratory Medicine and Center for Neurodegenerative Diseases Research, Perelman School of Medicine University of Pennsylvania (UPenn). They used the automated Roche Elecsys and cobas e immunoassay analyzer system. These measurements followed a Roche Study Protocol at the UPenn/ADNI Biomarker Laboratory, according to the preliminary kit manufacturer’s instructions and as described in previous studies [1, 16]. Data of plasma Aβ42/40 ratio were provided by the Bateman Lab using a high precision assay with the anti-Aβ mid-domain antibody on the automated immunoprecipitation platform (uploaded on June 2019).

### PET imaging acquisition and processing

Data of brain amyloid PET were acquired by the florbetapir (AV-45) tracer, and the summary data are regularly updated on the website of ADNI. The native-space MRI scans of participants were segmented by the Freesurfer (version 4.5.0). The mean uptakes of selected cortical and reference regions were then measured with the florbetapir scans which were applied to the MRI scans, correspondingly. Summary florbetapir standard uptake value ratios (SUVRs) were generated by averaging uptake ratios across four cortical regions (frontal, anterior/posterior cingulate, lateral parietal and lateral temporal regions) and then normalizing it by the reference region (whole cerebellum). Brain tau deposit was measured via the flortaucipir (AV-1451) processing method from the Helen Wills Neuroscience Institute, UC Berkeley and Lawrence Berkeley National Laboratory. We chose the composite metaROI of bilateral entorhinal, amygdala, fusiform, parahippocampal, inferior, and middle temporal regions for the tau PET evaluation [17]. To define the hypometabolic regions which were indicative of AD-related pathological metabolic change, FDG PET data were selected. A set of regions of interest (MetaROIs) were developed according to the literature review of FDG-PET studies on AD and MCI [18, 19]. The average counts of FDG PET across angular, temporal and posterior cingulate regions were adopted. Data of structural MRI was obtained by the Siemens Trio scanner and estimates of selected region volume were measured using Free-surfer software. The cutoffs for categories of brain amyloid, tau, and FDG PET were listed as below: 1.11 for florbetapir SUVR, 1.37 for flortaucipir metaROI SUVR [20], and 1.21 for FDG PET [18].

### Statistical analysis

All statistical analyses were conducted by R software (version 3.4.4). Characteristics of the cohort were presented as mean (standard deviation, SD) or number (percentage, %) when appropriate. Differences for continuous variables cross groups were assessed by the Kruskal-Wallis test and those for categorical data were evaluated by the chi-square test or Cochran–Mantel–Haenszel test. The Spearman’s rank correlation tested correlations of plasma p-tau181 with amyloid, tau, and FDG PET. The receiver operating curves (ROCs) comparing cohort subsets provided the area under the curves (AUCs) for a diagnosis of amyloid, tau, or FDG PET positivity. AUC, sensitivity, specificity, positive predictive value (PPV) and negative predictive value (NPV) at each optimal cutoff value were applied to assess the biomarker performance. Logistic regression model analyses were used when evaluating the diagnostic performance of plasma p-tau181 combined with clinical and genetic information (age, gender, years of education and *APOE* ɛ4 genotype). Differences between ROCs were tested by the DeLong’s test. ROC curves and the logistic regression (LR) analysis was carried out using the “OptimalCutpoints” and “pROC” packages in R. We also explored whether the relationships between plasma p-tau181 and PET biomarkers were mediated by CSF biomarkers, including Aβ_1-42_, total tau (t-tau), and p-tau. Mediation analyses were performed with the package “mediation” in R. Effects of CSF biomarkers and plasma p-tau181 on PET biomarkers were tested in the linear regression models, adjusted for age, gender, years of education and *APOE* ɛ4 genotype. Baron and Kenny proposed the methods which supported the linear regression models fitted [21]. For longitudinal analyses, baseline plasma p-tau181 were roughly dichotomized (PTAU+/PTAU−) by defining the cutoff that could best discriminate AD patients with Aβ-positivity from those normal subjects with Aβ-negativity. The ending events were defined as conversion from Aβ PET− to Aβ PET+, or conversion from FDG PET− to FDG PET+. In longitudinal analyses, we excluded borderline cases and reset the cutoffs that were ±5% from the original cutoffs to avoid drawing conclusions based on borderline cases [22, 23]. Unadjusted Kaplan–Meier plots were constructed to assess the risk of PTAU+/PTAU− groups progressing from PET biomarker negativity to positivity. Besides, we ran univariate and multivariate Cox proportional-hazards models to predict pathological progression. Age, gender, years of education and *APOE* ɛ4 genotype were included as covariates in the multivariate models. The statistical significance of all tests was set at a two-sided *P* value <0.05.

## Results

### Description of the study cohort

Demographic characteristics of the study cohort are summarized in Table 1, including clinical features and *APOE* genotype. A total of 1189 participants were enrolled, among which 1060 participants had data of amyloid PET, 195 had tau PET and 1085 had FDG PET (Table 1). Participants with abnormal uptake of PET tracer (PET+) presented to be older than those without (*P* < 0.05). The FDG-PET+ group had a larger proportion of male participants than the FDG-PET− group (*P* = 0.007), whereas amyloid-PET and tau-PET groups did not show significant difference in gender ratios. All PET+ groups showed significantly higher proportions of *APOE* ɛ4 carriers and poorer cognition performance, compared to the corresponding PET− groups (Table 1). Amyloid-PET+ and FDG-PET+ groups had more AD patients than PET− participants (28.0% versus 5.0%, and 38.9% versus 4.2%, respectively; *P* < 0.05, Table 1).

**Table 1:** Demographic features of the population in the present study.

	Aβ-PET -	Aβ-PET +	*P* value	Tau PET -	Tau PET +	*P* value	FDG-PET -	FDG-PET +	*P* value
N	500	560	-	171	24	-	671	414	-
Age, y	72.7 (7.7)	74.4 (7.2)	<0.001	71.5 (7.1)	74.6 (5.7)	0.024	72.7 (7.3)	75.3 (7.6)	<0.001
Gender, female	224 (44.8)	269 (48.0)	0.292	81 (47.4)	14 (58.3)	0.315	332 (49.5)	170 (41.1)	0.007
Education level, y	16.6 (2.5)	15.9 (2.8)	<0.001	16.5 (2.6)	16.0 (3.0)	0.435	16.3 (2.6)	15.9 (2.8)	0.044
*APOE* ɛ4 carriers	104 (20.8)	359 (64.1)	<0.001	60 (35.0)	14 (58.3)	0.033	234 (34.9)	240 (58.0)	<0.001
MMSE score	28.5 (2.0)	26.5 (3.3)	<0.001	28.4 (2.2)	26.3 (3.5)	<0.001	28.5 (1.9)	25.7 (3.6)	<0.001
CN/sMCI/pMCI/AD	245/222/8/25	116/218/69/157	<0.001	97/69/4/1	8/11/4/1	0.002	298/322/23/28	73/123/57/161	<0.001

Notes: Data are presented as mean (SD) or number (percentage) when appropriate. Abbreviations: Aβ, amyloid-β; PET, positron emission tomography; CN, cognitively normal; sMCI, stable mild cognitive impairment; pMCI, progressive mild cognitive impairment; AD, Alzheimer’s disease.

**Fig. 1:**
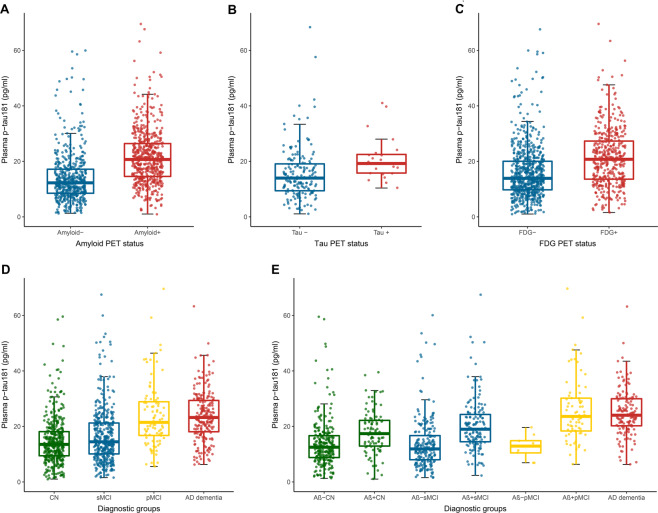
Plasma p-tau181 plots for PET and clinical classification groups. Box plots of plasma p-tau181 concentration between amyloid PET groups (**A**), tau PET groups (**B**), FDG PET groups (**C**), clinical diagnostic groups (**D**), or clinical classification combined with Aβ status (**E**). Raw data are presented on the box-and-whisker plot background. The middle line represents the median, and the upper and lower lines represent the first and third quartiles, respectively. Patients with mild cognitive impairment were excluded if they had a follow-up time less than 2 years. CN cognitively normal control, sMCI stable mild cognitive impairment, pMCI progressive mild cognitive impairment, AD Alzheimer’s disease.

Figure 1 demonstrated mean differences of plasma p-tau181 concentrations between PET groups and among diagnostic groups. Amyloid-PET+ participants had increased levels of plasma p-tau181 than amyloid-PET− individuals (21.6 ± 9.9 versus 14.4 ± 9.9 pg/ml, *P* < 0.0001; Fig. 1A). Tau-PET+ group showed a higher level of plasma p-tau181 than the tau-PET− group (20.9 ± 7.8 versus 15.5 ± 9.3 pg/ml, *P* = 0.0003; Fig. 1B). FDG-PET+ individuals also illustrated an elevated concentration compared with those with FDG-PET− (21.4 ± 10.3 versus 16.2 ± 10.1 pg/ml, *P* < 0.0001; Fig. 1C). AD patients (23.7 ± 8.8 pg/ml) and pMCI subjects (24.4 ± 15.4 pg/ml) had significantly higher levels of plasma p-tau181 than sMCI subjects (17.5 ± 10.9 pg/ml) and CN individuals (17.1 ± 25.4 pg/ml) (*P* < 0.001), whereas no difference was found between AD and pMCI patients (*P* > 0.05) (Fig. 1D). Considering amyloid status, individuals with Aβ-PET-positivity had increased plasma p-tau181 compared with those with Aβ-PET-negativity, separately in CN (18.0 ± 7.7 vs. 16.7 ± 30.2 pg/ml), sMCI (20.5 ± 9.7 vs. 13.9 ± 9.3 pg/ml), and pMCI (27.5 ± 16.3 vs. 12.6 ± 4.3 pg/ml) subgroups (*P* < 0.05; Fig. 1E).

### Relationships between plasma p-tau181 and PET biomarkers

**Fig. 2:**
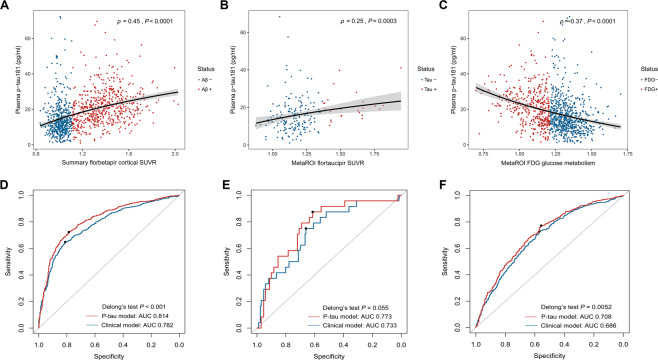
Relationships of plasma p-tau181 concentration with brain PET uptakes. Scatter fitting curves (**A**–**C**) plot the associations of plasma p-tau181 concentration with brain amyloid (**A**), tau (**B**), and FDG (**C**) PET uptakes. Statistical results were generated by the Spearman correlation analyses, and the corresponding curves were roughly smoothed to show directionality. Blue points represent plasma p-tau181 levels of individuals with normal PET uptakes, whereas red points represent those of individuals with abnormal PET uptakes. ROC curves (**D**–**F**) illustrate the performance of plasma p-tau181 in discriminating brain pathological changes (**D** for detecting the abnormal amyloid PET, **E** for detecting the abnormal tau PET, and **F** for detecting the abnormal FDG PET). The blue line represents the performance of the clinical model, consisting of age, gender, years of education, and *APOE* ɛ4 carriage. The red line represents the performance of the adjusted model which combined plasma p-tau181 and the clinical indicators including age, gender, years of education, and *APOE* ɛ4 carriage. ROC receiver operating characteristic; AUC area under the curve.

As shown in Fig. 2, plasma p-tau181 was positively correlated with the summary SUVRs of amyloid florbetapir tracer (Spearman *ρ* = 0.45, *P* < 0.0001; Fig. 2A) and tau flortaucipir tracer (Spearman *ρ* = 0.25, *P* = 0.0003; Fig. 2B). A significantly negative relationship was demonstrated between plasma p-tau181 and FDG glucose metabolism (Spearman *ρ* = −0.37, *P* < 0.0001; Fig. 2C). Furthermore, plasma p-tau181 correlated significantly with the cortical amyloid burden in Aβ-positive subjects (Spearman *ρ* = 0.30, *P* < 0.0001), while there was no statistical association in Aβ-negative subjects (Spearman *ρ* = −0.017, *P* = 0.72). Separately in subjects with or without abnormal tau deposition, no statistical correlations were observed between plasma p-tau181 concentration and metaROI flortaucipir SUVR (Spearman *ρ* = 0.11, *P* = 0.59; Spearman *ρ* = 0.14, *P* = 0.077, respectively). Among individuals with reduced glucose metabolism, plasma p-tau181 had an inverse link to metaROI FDG glucose metabolism (Spearman *ρ* = −0.31, *P* < 0.0001). This link tended to be slightly weaker in those without abnormal brain metabolism (Spearman *ρ* = −0.21, *P* < 0.0001).

### Discriminative accuracy of plasma p-tau181 for PET status

ROC analyses for plasma p-tau181 in discriminating brain pathologic changes presented by PET biomarkers were shown in Fig. 2. When we evaluated the diagnostic performance of plasma p-tau181, the ROC curves demonstrated an AUC of 0.755 (95%CI 0.721–0.781) for discriminating Aβ PET-positivity versus PET-negativity, an AUC of 0.728 (95%CI 0.642–0.814) for tau PET, and that of 0.672 (95%CI 0.639–0.705) for FDG PET (Supplementary). In the whole cohort sample, the adjusted models combining plasma p-tau181 and clinical information (age, gender, years of education, and *APOE* ɛ4 carriage) had moderately better performance for Aβ-PET positivity versus negativity than the model only containing clinical information (AUC 0.814 versus AUC 0.782, DeLong method test *P* < 0.001; Fig. 2D). Similar, albeit slightly weaker, performance was seen in predicting FDG PET status (AUC 0.708 versus AUC 0.686, DeLong method test *P* = 0.005; Fig. 2F). The comparison did not reach conventional statistical significance for tau PET status (AUC 0.773 versus AUC 0.733, DeLong method test *P* = 0.055; Fig. 2E). Concrete diagnostic performance (sensitivity, specificity, PPV, and NPV) of each individual and combined models were shown in the supplementary materials. Furthermore, we explored the performance of plasma p-tau181 combined with Aβ42/40 ratio in a subgroup of 183 subjects. Diagnostic accuracy had slight increments using the combined plasma markers (for Aβ PET status: AUC 0.701 vs. 0.786; for FDG PET status: AUC 0.607 vs. 0.642; no available data for tau PET). Given the small sample size, results of this subgroup should be interpreted with caution.

### CSF biomarkers statistically mediates the association between plasma p-tau181 and PET biomarkers

**Fig. 3:**
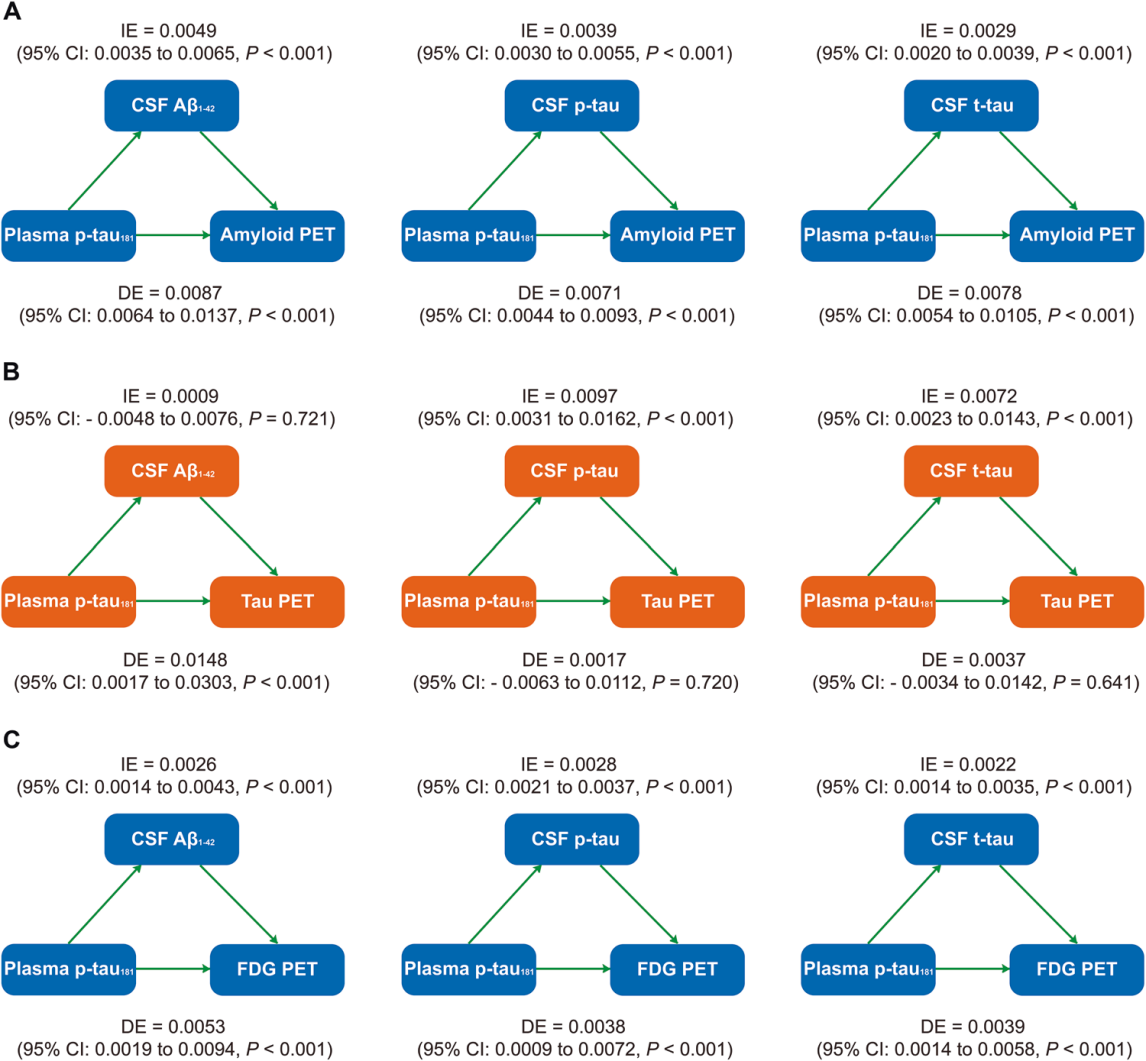
Mediation analyses with brain PET uptakes as pathological outcomes. The relationships of plasma p-tau181 with brain amyloid PET uptake (**A**), tau PET uptake (**B**), and FDG PET uptake (**C**) were mediated by CSF biomarkers. IE indirect effect, DE direct effect.

We performed mediation analyses to investigate whether plasma p-tau181 contributed to the prediction of Alzheimer’s pathologies assessed by PET via biomarkers in CSF. We found that CSF biomarkers, including Aβ_1–42_, p-tau, and t-tau, mediated the effect of plasma p-tau181 on amyloid PET with 29.6%, 30.4%, and 38.0% mediation, respectively (Fig. 3A). Using CSF p-tau or t-tau as the predictors, the effect of plasma p-tau181 on tau PET showed a full mediation (53.9% and 30.9%, respectively), as the effect was reduced from highly significant (*P* < 0.0001) to reach or near non-significance (*P* = 0.20, *P* = 0.046, respectively; Fig. 3B). However, there was no mediating effect of CSF Aβ_1–42_ (Fig. 3B). Similarly, these three kinds of CSF biomarkers all presented partial mediation for the influence of plasma p-tau181 on FDG PET, with moderate mediated proportions (28.8%, 30.4%, and 25.7%, respectively; Fig. 3C).

### Plasma p-tau181 predicts future amyloid and FDG PET progression

Following the findings that increased plasma p-tau181 appeared to correlated with changes in amyloid, tau, and FDG PET, we further tested whether it could predict the pathological progression in the brain over time. Subsets of individuals with normal amyloid, tau and FDG PET uptake were used to test whether abnormal plasma p-tau181 at baseline could predict a subsequent deterioration in pathologies. Participants with eligible plasma data and clinical diagnosis were enrolled for evaluation of the cutoff point for plasma p-tau181 after the procedures of quality control. Descriptive characteristics of this cohort were described in the Supplementary. ROC analyses of Aβ-positive AD patients versus Aβ-negative normal controls provided the statistically optimal cutoff for plasma p-tau181 (Supplementary). The cutoff for plasma p-tau181 was 18.85 pg/ml with an AUC of 0.840 (95%CI 0.80–0.88; sensitivity 81.1% and specificity 81.6%). This cutoff value had a promising negative predictive value of 86.4%, and a logistic regression AUC of 0.850 after adjusting for age, gender, years of education and *APOE* genotype.

**Fig. 4:**
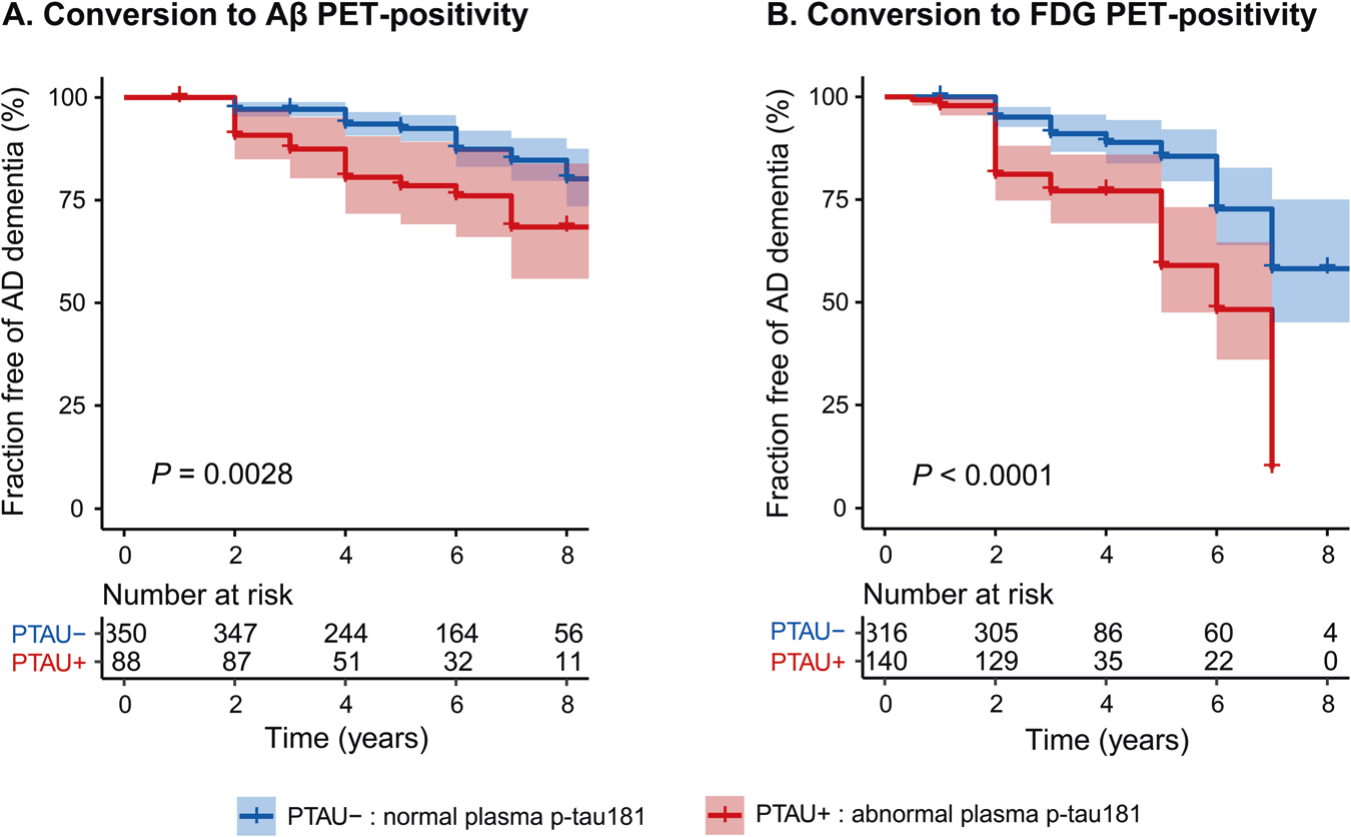
Relationships of baseline plasma p-tau181 level with the risk of pathological progression. Unadjusted Kaplan–Meier plots show the risk of conversion from amyloid PET negative to amyloid PET positive (**A**), and from FDG PET negative to FDG PET positive (**B**) between baseline plasma p-tau181 status. PTAU−, individuals with baseline plasma p-tau181 concentration less than 18.85 pg/ml; PTAU+, individuals with baseline plasma p-tau181 concentration more than 18.85 pg/ml.

Participants with normal PET uptake at baseline were included and categorized into two groups by the cut-point of plasma p-tau181 (18.85 pg/ml). A subcohort of 438 individuals who had normal Aβ PET at baseline underwent at least once Aβ PET scan during follow-up periods. Fifty-four subjects (12.3%) converted to Aβ PET-positive over the follow-up period while others remained negative. Among these, individuals with baseline abnormal p-tau181 in the plasma (PTAU+) had a higher risk of conversion from Aβ PET-negativity to PET-positivity (*P* = 0.0028, Fig. 4A), compared with those with normal plasma levels of p-tau181 at baseline (PTAU−). PTAU+ individuals had a larger proportion rate than the PTAU− group (20.5% vs. 10.3%, Supplementary). A subset of 456 individuals were FDG PET-normal at baseline, of which 81 (17.8%) converted to FDG PET abnormality during the follow-up period. PTAU+ subjects showed an increased risk of conversion to FDG PET abnormality compared with PTAU− individuals (*P* < 0.0001, Fig. 4B; conversion rate: 30.7% vs. 12.0%, Supplementary).

## Discussion

In the present study, we aimed to evaluate the potential role of plasma p-tau181 in predicting brain amyloid, tau, and neurodegeneration pathologies presented by the PET technique. We found that the plasma levels of p-tau181 elevated in response to the deterioration of brain pathological changes in the process of AD, which were partly or entirely mediated by CSF biomarkers. Longitudinally, plasma p-tau181 could serve as a progression marker for the Alzheimer’s pathological processes.

Our data comprehensively demonstrated that elevated p-tau181 levels in the periphery were obviously correlated with Alzheimer’s pathological changes in the brain, including abnormal amyloid accumulation, tau deposition and neurodegeneration presented by the decreased glucose metabolism in the brain. Participants with abnormal amyloid, tau burden and hypometabolism in the brain had significantly higher levels of plasma p-tau181, in comparison to those who had normal PET uptakes. Similar findings were proved in other studies which tested plasma biomarkers including p-tau181 [10, 14, 24]. But differently, they lack the evaluation of the possible relationship between plasma p-tau181 and brain neurodegeneration, or chose cortical thickness as the predictor for neuronal injury or neurodegeneration, and reported diverse outcomes. Mielke et al. did not find the possible correlation of plasma p-tau181 with cortical thickness in the AD-featured brain regions [10], whereas the recent study suggested that peripheral p-tau181 level predicted the hippocampal atrophy during a 1-year follow-up period [14]. In our cohort, plasma p-tau181 not only had a cross-sectional relationship with brain neurodegeneration measured by glucose metabolism of metaROIs, but also served as a predictor for neurodegenerative progression. This finding replenished and validated the potential role of plasma p-tau181 as a biomarker for Alzheimer’s pathologies in the brain.

Plasma p-tau181 increased already in the preclinical and prodromal stages of AD prior to the clinical cognitive deterioration, even when measuring plasma samples collected ahead of negative tau PET scans. It has been previously proved that plasma p-tau181 elevated along the Alzheimer’s continuum from preclinical stage to clinical dementia phase [25, 26]. In the present cohort, we categorized the MCI patients into stable MCI (sMCI) and progressive MCI (pMCI) subgroups by whether they conversed to AD dementia in a two-year follow-up period. The clinical subdivision of MCI individuals helped to define the exact distribution of plasma p-tau181 across diagnostic groups. AD and pMCI patients had significantly higher levels of plasma p-tau181 than sMCI patients and cognitively normal controls. However, no difference was found between AD patients and pMCI individuals. Thus, it was reasonable to suspect that the pathological increment of plasma p-tau181 occurred in the early stages of the AD continuum, prior to the clinical cognitive deterioration. To further explore this, we added brain Aβ pathology as a stratification factor for that it was acknowledged as the most featured pathology of AD. In both MCI patients and cognitively normal controls, Aβ-positive individuals proved to have obviously increased levels of plasma p-tau181 than Aβ-negative individuals. Also, MCI patients with Aβ-positivity possessed an increment of peripheral p-tau181 compared to cognitively normal controls with Aβ-positivity. Other cohorts also explored the differences of plasma tau measures by both clinical diagnosis and Aβ PET [10, 14, 27], but they lacked analysis stratified by clinical conversion and observed no statistical difference between the CU A+ and A− groups or the CU and MCI A+ groups [10]. In total, PET-positive individuals all showed significantly elevated concentrations of plasma p-tau181, with approximate increments of 30–50% compared to PET-negative groups. These observations also implemented recent findings showing that plasma biomarker profiles including p-tau181 could predict clinical progression in preclinical and prodromal patients [25].

Both individually and combined with clinical indicators, plasma p-tau181 significantly detected amyloid, tau and neurodegeneration pathologies in the brain. In this cohort, we found the relatively good performance of plasma p-tau181 for distinguishing abnormal uptakes of Aβ, tau and FDG PET from the normal. These performances were similar to or even better than the combined diagnostic capacity of age, gender, education and *APOE* genotype in discriminating PET abnormality from the normal. Moreover, the combined model consisting of both plasma p-tau181 and clinical indicators proved to be more accurate for Alzheimer’s pathological definition than the model which merely had clinical information. In our cohort, the predictive accuracy of plasma p-tau181 for increased brain amyloid burden was very close to that from the Mayo Clinic data [10], suggesting a stable discriminative performance of plasma p-tau181 in different cohorts. We also found that plasma p-tau181 could detect abnormal tau deposition with moderate accuracy. This finding was in line with the recent studies, which investigated the usefulness of plasma p-tau181 for differential diagnosis [13, 25]. Janelidze et al. conducted ROC analyses separately in Braak I-IV ROI and Braak V-VI ROI [28]. Compared with our cohort, their study reported slightly better diagnostic performance of plasma p-tau181 in the Braak I-IV ROI (AUC 0.86, 95%CI 0.80–0.92) which tends to represent the early stage of tau pathology process in AD [28]. And the diagnostic capacity demonstrated to grow even larger in the Braak V-VI ROI (AUC 0.90, 95%CI 0.84–0.96) which indicated more severe tau pathology [28]. The diagnostic performance of plasma p-tau181 in our cohort was similar to that of Park et al., with both high sensitivity (AUC 0.731, sensitivity 93.33%) [13]. Besides, plasma p-tau181 showed moderate diagnostic ability in detecting hypometabolism of metaROIs in the brain. When combined with plasma β-amyloid, the diagnostic performance of plasma p-tau181 had only slight increase in detecting cerebral Alzheimer’s pathologies. These results were similar to a previous study, which provided that combination of plasma tau and β-amyloid might be slightly better in predicting brain tau pathology and neurodegeneration [13]. These findings indicated that though single plasma p-tau181 might not be the most preferred approach for AD, it was highly efficient in detecting AD pathology. However, considering the small sample size in both ours and the previous study [13], comparisons of plasma p-tau181 and its combination with other plasma markers are needed to be explored in more large, multi-centered studies in the future. Additionally, in part or in whole, we found that pathological changes of amyloid, tau in CSF outstandingly mediated the role of peripheral p-tau181 in detecting Alzheimer’s pathologies in the brain. These findings also supported the previously published points of view that the breakdown or dysfunction of the blood-brain barrier was associated with the pathogenesis of complex multifactorial diseased which included AD [29, 30]. All these findings indicated an exciting role of peripheral p-tau181 for detecting Alzheimer’s pathologies in the central nervous system.

Furthermore, our previous study has demonstrated that the plasma p-tau181 could be utilized as a progression marker for clinical conversion and cognitive decline [31], and the present findings proved that it was also specific for the AD pathophysiological processes. Individuals with abnormal plasma p-tau181 levels were at higher risk of deterioration of brain amyloidosis and neurodegeneration exceeding the critical boundaries. Due to long time intervals between plasma samples and tau PET scans in the vast majority of participants, survival analyses were not conducted for tau PET conversion. Additionally in a recent study by our team, we observed that plasma p-tau181 increased on the single patient level over time as the disease progressed [32]. It was also confirmed that plasma p-tau181 was longitudinally correlated with AD-related CSF and PET neuroimaging biomarkers, both in the whole population or in subgroups defined by ATN classifications and cognitive status [32]. Together, these findings supported the effectiveness of plasma p-tau181 in monitoring pathological changes during the course of AD. Previous studies mainly focused on the conversion of clinical outcomes [25], but lack further insight into the role of plasma p-tau181 in predicting the pathological changes of AD. Our study filled the gap in this area, and suggesting that the plasma p-tau181 had the potential to serve as a sufficient biomarker for tracking the Alzheimer’s pathological process. Future studies on tau PET are warranted to expand validation results.

The present study has limitations. Though plasma biomarker profiles have advantages over PET or CSF measurements, at present, advance in this field was confined due to different experimental methods, clinical population across laboratories and lack of reproductivity. Our study cohort did not have individuals due to non-AD dementia, which hampered the assessments of plasma p-tau181 in discriminating AD versus other kinds of dementia [3, 11]. There also lack sufficient longitudinal data to further evaluate the potential role of plasma p-tau181 for predicting brain tau deposits measured by PET. Besides in the present cohort, only 24 subjects were defined as tau PET-positivity, which limited the precise evaluation in detecting brain tau deposition. Given the small sample size, more comprehensive research in larger cohorts are required to define the diagnostic potential of plasma p-tau181 for brain tau pathology.

In total, we suggested that plasma p-tau181 could be utilized as the preliminary and practical test for the detection of Alzheimer’s pathology. This measurement could help ruling out the pathological definition of AD, and guide the population screening and therapy monitoring for disease-modifying trials. Future validation research is demanded in larger, more diverse population to confirm these findings and provide more insight into this plasma biomarker over longer periods of disease progression.

## Supplementary information

Supplementary

## Data Availability

Data used in the preparation of this article were obtained from the ADNI database (adni.loni.usc.edu), which is easily available for the research public.
