# Supplementary material for: Plasma phosphorylated-tau181 as a predictive biomarker for Alzheimer’s amyloid, tau and FDG PET status
Source: Transl Psychiatry. 2021 Nov 13;11:585. doi: 10.1038/s41398-021-01709-9 (PMC8590691; doi:10.1038/s41398-021-01709-9)
Supplement: Supplementary file 1 — Supplementary [file 41398_2021_1709_MOESM1_ESM.docx]

**Supplementary**

**eFigure 1.** ROC curve of plasma p-tau181 for diagnosing Aβ+ AD patients from Aβ- cognitively controls

**eFigure 2.** Logistic regression model ROC curve of plasma p-tau181 for diagnosing Aβ+ AD patients from Aβ- cognitively controls

**eTable 1.** Diagnostic performance of individual and combined models in detecting brain Aβ, tau and FDG PET status

**eTable 2.** Pathological progression risk of participants grouped by different plasma p-tau181 level at baseline

**The member list of Alzheimer’s Disease Neuroimaging Initiative (ADNI)**

**eFigure 1. ROC curve of plasma p-tau181 for diagnosing Aβ+ AD patients from Aβ- cognitively controls**

**
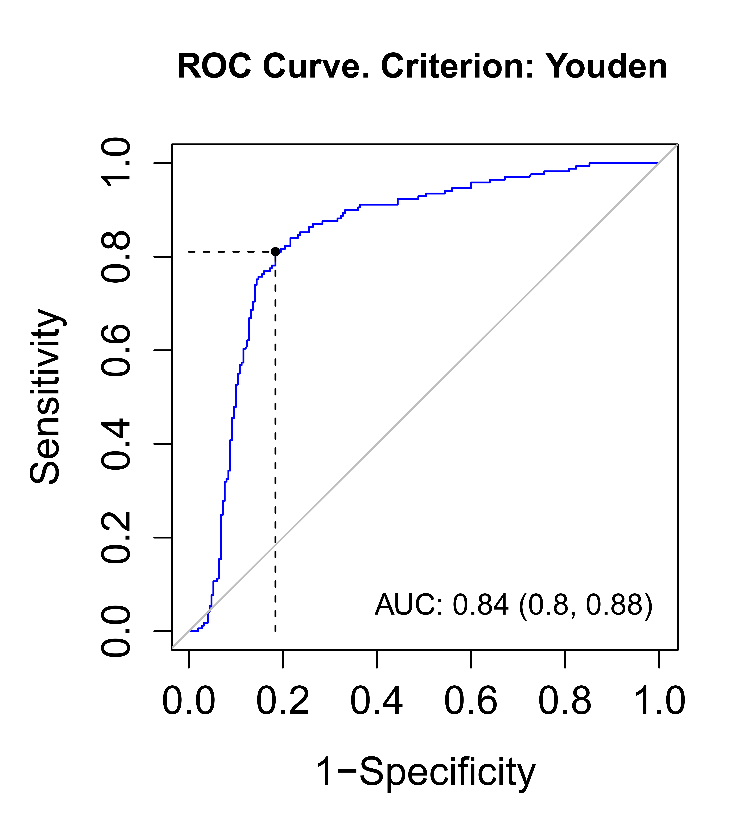
**

**Abbreviation:** ROC, receiver operating curve; Aβ, amyloid-β; AD, Alzheimer’s disease.

**eFigure 2. Logistic regression model ROC curve of plasma p-tau181 for diagnosing Aβ+ AD patients from Aβ- cognitively controls**

**
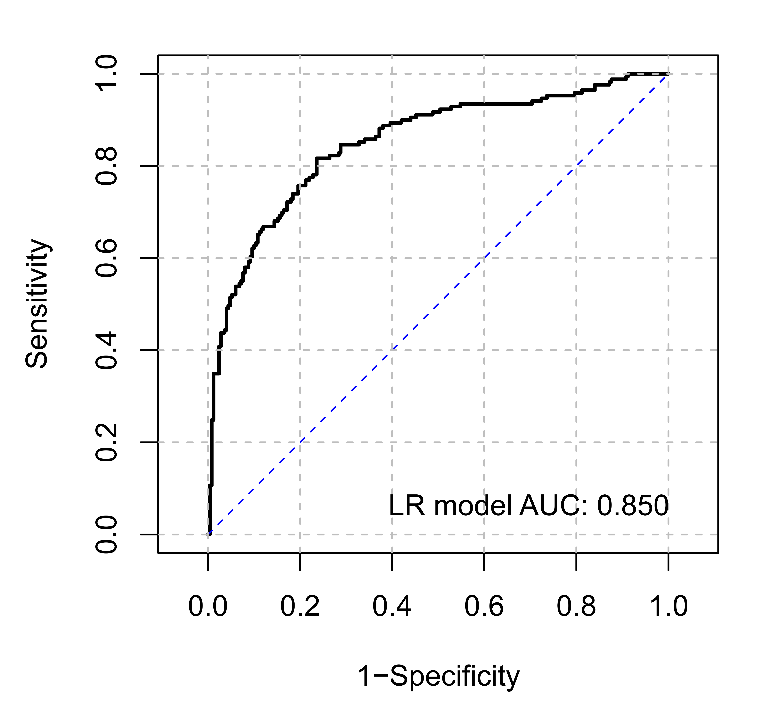
**

**Note:** The logistic regression model ROC curve combined plasma p-tau181 and clinical/genetic information, including age, gender, years of education, and *APOE* ɛ4 carriage.

**Abbreviation:** LR, logistic regression; ROC, receiver operating curve; Aβ, amyloid-β; AD, Alzheimer’s disease.

| **eTable 1. Diagnostic performance of individual and combined models in detecting brain Aβ, tau and FDG PET status** | | | | | | |
| --- | --- | --- | --- | --- | --- | --- |
|  | | AUC | Sensitivity | Specificity | PPV | NPV |
| Detecting brain Aβ PET positivity (n = 1060) | |  |  |  |  |  |
|  | Plasma p-tau | 0.755 | 0.766 | 0.650 | 0.710 | 0.713 |
|  | Age+*APOE* genotype+clinical model | 0.782 | 0.646 | 0.814 | 0.796 | 0.673 |
|  | P-tau+Age+*APOE* genotype+clinical model | 0.814 | 0.755 | 0.756 | 0.776 | 0.734 |
| Detecting brain tau PET positivity (n = 195) | |  |  |  |  |  |
|  | Plasma p-tau | 0.728 | 0.833 | 0.573 | 0.215 | 0.961 |
|  | Age+*APOE* genotype+clinical model | 0.733 | 0.875 | 0.602 | 0.236 | 0.972 |
|  | P-tau+Age+*APOE* genotype+clinical model | 0.773 | 0.875 | 0.614 | 0.241 | 0.972 |
| Detecting brain FDG PET positivity (n = 1085) | |  |  |  |  |  |
|  | Plasma p-tau | 0.672 | 0.592 | 0.705 | 0.553 | 0.737 |
|  | Age+*APOE* genotype+clinical model | 0.686 | 0.751 | 0.720 | 0.623 | 0.824 |
|  | P-tau+Age+*APOE* genotype+clinical model | 0.708 | 0.713 | 0.627 | 0.541 | 0.780 |

Abbreviation: Aβ, amyloid-β; PPV, positive predictive value; NPV, negative predictive value.

| **eTable 2. Pathological progression risk of participants grouped by different plasma p-tau181 level at baseline** | | | | | | | |
| --- | --- | --- | --- | --- | --- | --- | --- |
|  | | Conversion rate | Unadjusted | |  | Adjusted | |
|  |  |  | Hazard ratio (95% CI) ^a^ | *P* value |  | Hazard ratio (95% CI) ^b^ | *P* value |
| Conversion from Aβ PET- to Aβ PET+ | | | | | | | |
|  | PTAU- | 10.3% | ref. | - |  | ref. | - |
|  | PTAU+ | 20.5% | 2.32 (1.32 - 4.08) | .004 |  | 2.20 (1.22 - 3.94) | .008 |
| Conversion from FDG PET- to FDG PET+ | | | | | | | |
|  | PTAU- | 12.0% | ref. | - |  | ref. | - |
|  | PTAU+ | 30.7% | 3..21 (2.06 - 5.01) | < .001 |  | 2.66 (1.67 - 4.23) | < .001 |

Abbreviation: Aβ, amyloid-β; PTAU, plasma phosphorylated-tau181; PTAU-, individuals who had plasma p-tau181 level less than 18.85 pg/ml at baseline; PTAU+, individuals who had plasma p-tau181 level more than 18.85 pg/ml at baseline.

^a^ Hazard ratio (95% CI) calculated using Cox regression analyses.

^b^ Hazard ratio (95% CI) calculated using Cox regression analyses and corrected for baseline age, gender, *APOE* ε4 status and years of education.

**The member list of Alzheimer’s Disease Neuroimaging Initiative (ADNI)**

Michael W. Weiner, MD ^1^, Paul Aisen, MD ^2^, Ronald Petersen, MD, PhD ^3^, Clifford R. Jack, Jr., MD ^3^, William Jagust, MD ^4^, John Q. Trojanowki, MD, PhD ^5^, Arthur W. Toga, PhD ^2^, Laurel Beckett, PhD ^6^, Robert C. Green, MD, MPH ^7^, Andrew J. Saykin, PsyD ^8^, John C. Morris, MD ^9^, Richard J. Perrin, MD, PhD ^9^, Leslie M. Shaw, PhD ^5^, Maria Carrillo, PhD ^10^, William Potter, MD ^11^, Lisa Barnes, PhD ^12^, Marie Bernard, MD ^13^, Hector González ^14^, Carole Ho ^15^, John K. Hsiao, MD ^16^, Jonathan Jackson, PhD ^17^, Eliezer Masliah, MD ^13^, Donna Masterman, MD ^18^, Ozioma Okonkwo, PhD ^19^, Richard Perrin, MD ^20^, Laurie Ryan, PhD ^13^, Nina Silverberg, PhD ^13^, Adam Fleisher, MD ^21^, Michael W. Weiner, MD ^1^, Diana Truran Sacrey, ^22^, Juliet Fockler, ^1^, Cat Conti, BA ^22^, Dallas Veitch, PhD ^22^, John Neuhaus, PhD ^1^, Chengshi Jin, PhD ^1^, Rachel Nosheny, PhD ^1^, Miriam Ashford, PhD ^22^, Derek Flenniken, ^22^, Adrienne Kormos, ^22^, Cat Conti, BA ^22^, Paul Aisen, MD ^2^, Michael Rafii, MD, PhD ^2^, Rema Raman, PhD ^2^, Gustavo Jimenez, MBS ^2^, Michael Donohue, PhD ^2^, Devon Gessert, BS ^2^, Jennifer Salazar, MBS ^2^, Caileigh Zimmerman, MS ^2^, Yuliana Cabrera, BS ^2^, Sarah Walter, MSc ^2^, Garrett Miller, MS ^2^, Godfrey Coker, MBA, MPH ^2^, Taylor Clanton, MPH ^2^, Lindsey Hergesheimer, BS ^2^, Stephanie Smith, BS ^2^, Olusegun Adegoke, MSc ^2^, Payam Mahboubi, MPH ^2^, Shelley Moore, BA ^2^, Jeremy Pizzola, BA ^2^, Elizabeth Shaffer, BS ^2^, Brittany Sloan, BA ^2^, Danielle Harvey, PhD ^6^, Michael Donohue, PhD ^2^, Arvin Forghanian-Arani, PhD ^23^, Bret Borowski, RTR ^23^, Chad Ward, ^23^, Christopher Schwarz, PhD ^23^, David Jones, MD ^23^, Jeff Gunter, PhD ^23^, Kejal Kantarci, MD ^23^, Matthew Senjem, MS ^23^, Prashanthi Vemuri, PhD ^23^, Robert Reid, PhD ^23^, Nick C. Fox, MD ^24^, Ian Malone, PhD ^24^, Paul Thompson, PhD ^25^, Sophia I. Thomopoulos, BS ^25^, Talia M. Nir, PhD ^25^, Neda Jahanshad, PhD ^25^, Charles DeCarli, MD ^6^, Alexander Knaack, MS ^6^, Evan Fletcher, PhD ^6^, Duygu Tosun-Turgut, PhD ^1^, Stephanie Rossi Chen, BA. ^22^, Mark Choe, BS ^22^, Karen Crawford, ^25^, Paul A. Yushkevich, PhD ^5^, Sandhitsu Das, PhDMayo Clinic ^5^, Robert A. Koeppe, PhD ^26^, Eric M. Reiman, MD ^27^, Kewei Chen, PhD ^27^, Chet Mathis, MD ^28^, Susan Landau, PhD ^4^, Richard Perrin MD ^9^, Nigel J. Cairns, PhD, FRCPath ^9^, Erin Householder, MS ^9^, Erin Franklin, MS ^9^, Haley Bernhardt, BA, R. EEG T ^9^, Lisa Taylor-Reinwald, BA, HTL ^9^, Leslie M. Shaw, PhD ^29^, John Q. Trojanowki, MD, PhD ^29^, Magdalena Korecka, PhD ^29^, Michal Figurski, PhD ^29^, Karen Crawford ^2^, Scott Neu, PhD ^2^, Andrew J. Saykin, PsyD ^30^, Kwangsik Nho, PhD ^30^, Shannon L. Risacher, PhD ^30^, Liana G. Apostolova, MD ^30^, Li Shen, PhD ^31^, Tatiana M. Foroud, PhD ^32^, Kelly Nudelman, PhD ^32^, Kelley Faber, MS, CCRC ^32^, Kristi Wilmes, MS, CCRP ^32^, Leon Thal, MD – Past Investigator ^14^, Zaven Khachaturian, PhD ^33^, John K. Hsiao, MD ^34^, Lisa C. Silbert, MD ^35^, Betty Lind, BS ^35^, Rachel Crissey ^35^, Jeffrey A. Kaye, MD, A ^35^, Raina Carter, BA ^35^, Sara Dolen, BS ^35^, Joseph Quinn, MD ^35^, Lon S. Schneider, MD ^2^, Sonia Pawluczyk, MD ^2^, Mauricio Becerra, MD ^2^, Liberty Teodoro, RN ^2^, Karen Dagerman, MS ^2^, Bryan M. Spann, DO, PhD ^2^, James Brewer, MD, PhD ^14^, Helen Vanderswag, RN ^36^, Adam Fleisher, MD ^36^, Jaimie Ziolkowski, MA, BS, TLLP ^26^, Judith L. Heidebrink, MD, MS ^26^, Lisa Zbizek-Nulph, MS ^26^, Joanne L. Lord, LPN, BA, CCRC ^26^, Lisa Zbizek-Nulph, MS, CCRP ^26^, Sara S. Mason, RN ^3^, Colleen S. Albers, RN ^3^, David Knopman, MD ^3^, Kris Johnson, RN ^3^, Javier Villanueva-Meyer, MD ^37^, Valory Pavlik, PhD ^37^, Nathaniel Pacini, MA ^37^, Ashley Lamb, MA ^37^, Joseph S. Kass, MD, LD, FAAN ^37^, Rachelle S. Doody, MD, PhD ^37^, Victoria Shibley, MS ^37^, Munir Chowdhury, MBBS, MS ^37^, Susan Rountree, MD ^37^, Mimi Dang, MD ^37^, Yaakov Stern, PhD ^38^, Lawrence S. Honig, MD, PhD ^38^, Akiva Mintz, MD, PhD ^38^, Beau Ances, MD, PhD, MSc ^39^, David Winkfield, BS ^39^, Maria Carroll, RN, MSN, GCNS-BC ^39^, Georgia Stobbs-Cucchi, RN, CCRP ^39^, Angela Oliver, RN, BSN, MSG ^39^, Mary L. Creech, RN, MSW ^39^, Mark A. Mintun, MD ^39^, Stacy Schneider, APRN, BC, GNP ^39^, David Geldmacher, MD ^40^, Marissa Natelson Love, MD ^40^, Randall Griffith, PhD, ABPP ^40^, David Clark, MD ^40^, John Brockington, MD ^40^, Daniel Marson, JD, PhD ^40^, Hillel Grossman, MD ^41^, Martin A. Goldstein, MD ^41^, Jonathan Greenberg, BA ^41^, Effie Mitsis, PhD ^41^, Raj C. Shah, MD ^42^, Melissa Lamar, PhD ^42^, Patricia Samuels ^42^, Ranjan Duara, MD ^43^, Maria T. Greig-Custo, MD ^43^, Rosemarie Rodriguez, PhD ^43^, Marilyn Albert, PhD ^44^, Chiadi Onyike, MD ^44^, Leonie Farrington, RN ^44^, Scott Rudow, BS ^44^, Rottislav Brichko, BS ^44^, Stephanie Kielb, BS ^44^, Amanda Smith, MD ^45^, Balebail Ashok Raj, MD ^45^, Kristin Fargher, MD ^45^, Martin Sadowski, MD, PhD ^46^, Thomas Wisniewski, MD ^46^, Melanie Shulman, MD ^46^, Arline Faustin, MD ^46^, Julia Rao, PhD ^46^, Karen M. Castro, BA ^46^, Anaztasia Ulysse, BA ^46^, Shannon Chen, BA ^46^, Mohammed O. Sheikh, MD ^46^, Jamika Singleton-Garvin, CCRP ^46^, P. Murali Doraiswamy, MBBS, FRCP ^47^, Jeffrey R. Petrella, MD ^47^, Olga James, MD ^47^, Terence Z. Wong, MD ^47^, Salvador Borges-Neto, MD ^47^, Jason H. Karlawish, MD ^5^, David A. Wolk, MD ^5^, Sanjeev Vaishnavi, MD ^5^, Christopher M. Clark, MD ^5^, Steven E. Arnold, MD ^5^, Charles D. Smith, MD ^48^, Gregory A. Jicha, MD, PhD ^48^, Riham El Khouli, MD ^48^, Flavius D. Raslau, MD ^48^, Oscar L. Lopez, MD ^28^, MaryAnn Oakley, MA ^28^, Donna M. Simpson, CRNP, MPH ^28^, Anton P. Porsteinsson, MD ^49^, Kim Martin, RN ^49^, Nancy Kowalski, MS, RNC ^49^, Melanie Keltz, RN ^49^, Bonnie S. Goldstein, MS, NP  ^49^, Kelly M. Makino, BS ^49^, M. Saleem Ismail, MD ^49^, Connie Brand, RN  ^49^, Gaby Thai, MD ^50^, Aimee Pierce, MD ^50^, Beatriz Yanez, RN ^50^, Elizabeth Sosa, PhD ^50^, Megan Witbracht, PhD ^50^, Brendan Kelley, MD ^51^, Trung Nguyen, MD ^51^, Kyle Womack, MD ^51^, Dana Mathews, MD, PhD ^51^, Mary Quiceno, MD ^51^, Allan I. Levey, MD, PhD ^52^, James J. Lah, MD, PhD ^52^, Ihab Hajjar, MD ^52^, Janet S. Cellar, DNP, PMHCNS-BC ^52^, Jeffrey M. Burns, MD ^53^, Russell H. Swerdlow, MD ^53^, William M. Brooks, PhD ^53^, Daniel H.S. Silverman, MD, PhD ^54^, Sarah Kremen, MD ^54^, Liana Apostolova, MD ^54^, Kathleen Tingus, PhD ^54^, Po H. Lu, PsyD ^54^, George Bartzokis, MD ^54^, Ellen Woo, PhD ^54^, Edmond Teng, MD, PhD ^54^, Neill R Graff-Radford, MBBCH, FRCP ^55^, Francine Parfitt, MSH, CCRC ^55^, Kim Poki-Walker, BA ^55^, Martin R. Farlow, MD ^8^, Ann Marie Hake, MD ^8^, Brandy R. Matthews, MD ^8^, Jared R. Brosch, MD ^8^, Scott Herring, RN, CCRC ^8^, Christopher H. van Dyck, MD ^56^, Adam P. Mecca, MD, PhD ^56^, Susan P. Good, APRN ^56^, Martha G. MacAvoy, PhD ^56^, Richard E. Carson, PhD ^56^, Pradeep Varma, MD ^56^, Howard Chertkow, MD ^57^, Susan Vaitekunis, MD ^57^, Chris Hosein, MEd ^57^, Sandra Black, MD, FRCPC ^58^, Bojana Stefanovic, PhD ^58^, Chris (Chinthaka) Heyn, BSC, PhD, MD, FRCPC ^58^, Ging-Yuek Robin Hsiung, MD, MHSc, FRCPC ^59^, Ellen Kim, BA ^59^, Benita Mudge, BS ^59^, Vesna Sossi, PhD ^59^, Howard Feldman, MD, FRCPC ^59^, Michele Assaly, MA ^59^, Elizabeth Finger, MD ^60^, Stephen Pasternak, MD ^60^, Irina Rachinsky, MD ^60^, Andrew Kertesz, MD ^60^, Dick Drost, MD ^60^, John Rogers, MD ^60^, Ian Grant, MD ^61^, Brittanie Muse, MSPH ^61^, Emily Rogalski, PhD ^61^, Jordan Robson ^61^, M.-Marsel Mesulam, MD ^61^, Diana Kerwin, MD ^61^, Chuang-Kuo Wu, MD, PhD ^61^, Nancy Johnson, PhD ^61^, Kristine Lipowski, MA ^61^, Sandra Weintraub, PhD ^61^, Borna Bonakdarpour, MD ^61^, Nunzio Pomara, MD ^62^, Raymundo Hernando, MD ^62^, Antero Sarrael, MD ^62^, Howard J. Rosen, MD ^1^, Bruce L. Miller, MD ^1^, David Perry, MD ^1^, Raymond Scott Turner, MD, PhD ^63^, Kathleen Johnson, NP ^63^, Brigid Reynolds, NP ^63^, Kelly MCCann, BA ^63^, Jessica Poe, BS ^63^, Reisa A. Sperling, MD ^64^, Keith A. Johnson, MD ^64^, Gad A. Marshall, MD ^64^, Christine M. Belden, PsyD ^65^, Alireza Atri, MD, PhD ^65^, Bryan M. Spann, DO, PhD ^65^, Kelly A. Clark ^65^, Edward Zamrini, MD ^65^, Marwan Sabbagh, MD ^65^, Ronald Killiany, PhD ^66^, Robert Stern, PhD ^66^, Jesse Mez, MD, MS ^66^, Neil Kowall, MD ^66^, Andrew E. Budson, MD ^66^, Thomas O. Obisesan, MD, MPH ^67^, Oyonumo E. Ntekim, MD, PhD ^67^, Saba Wolday, MSc ^67^, Javed I. Khan, MD ^67^, Evaristus Nwulia, MD ^67^, Sheeba Nadarajah, PhD ^67^, Alan Lerner, MD ^68^, Paula Ogrocki, PhD ^68^, Curtis Tatsuoka, PhD ^68^, Parianne Fatica, BA, CCRC ^68^, Evan Fletcher, PhD ^69^, Pauline Maillard, PhD ^69^, John Olichney, MD ^69^, Charles DeCarli, MD ^69^, Owen Carmichael, PhD ^69^, Vernice Bates, MD ^70^, Horacio Capote, MD ^70^, Michelle Rainka, PharmD, CCRP ^70^, Michael Borrie, MB ChB ^71^, T-Y Lee, PhD ^71^, Dr Rob Bartha, PhD ^71^, Sterling Johnson, PhD ^72^, Sanjay Asthana, MD ^72^, Cynthia M. Carlsson, MD, MS ^72^, Allison Perrin, PhD ^73^, Anna Burke, PhD ^73^, Douglas W. Scharre, MD ^74^, Maria Kataki, MD, PhD ^74^, Rawan Tarawneh, MD ^74^, Brendan Kelley, MD ^74^, David Hart, MD ^75^, Earl A. Zimmerman, MD ^75^, Dzintra Celmins, MD ^75^, Delwyn D. Miller, PharmD, MD ^76^, Laura L. Boles Ponto, PhD ^76^, Karen Ekstam Smith, RN ^76^, Hristina Koleva, MD ^76^, Hyungsub Shim, MD ^76^, Ki Won Nam, MD ^76^, Susan K. Schultz, MD ^76^, Jeff D. Williamson, MD, MHS ^77^, Suzanne Craft, PhD ^77^, Jo Cleveland, MD ^77^, Mia Yang, MD– Past Investigator ^77^, Kaycee M. Sink, MD, MAS ^77^, Brian R. Ott, MD ^78^, Jonathan Drake, MD ^78^, Geoffrey Tremont, PhD ^78^, Lori A. Daiello, Pharm.D, ScM ^78^, Jonathan D. Drake, MD ^78^, Marwan Sabbagh, MD ^79^, Aaron Ritter, MD ^79^, Charles Bernick, MD, MPH ^79^, Donna Munic, PhD ^79^, Akiva Mintz, MD, PhD ^79^, Abigail O’Connelll, MS, APRN, FNP-C ^80^, Jacobo Mintzer, MD, MBA ^80^, Arthur Wiliams, BS ^80^, Joseph Masdeu, PhD ^81^, Jiong Shi, MD, PhD ^82^, Angelica Garcia, BS ^82^, Marwan Sabbagh ^82^, Paul Newhouse, PhD ^83^, Steven Potkin, PhD ^84^, Stephen Salloway, MD, MS ^85^, Paul Malloy, PhD ^85^, Stephen Correia, PhD ^85^, Smita Kittur, MD ^86^, Godfrey D. Pearlson, MD ^87^, Karen Blank, MD ^87^, Karen Anderson, RN ^87^, Laura A. Flashman, PhD ^88^, Marc Seltzer, MD ^88^, Mary L. Hynes, RN, MPH ^88^, Robert B. Santulli, MD ^88^, Norman Relkin, MD, PhD  ^89^, Gloria Chiang, MD ^89^, Michael Lin, MD ^89^, Lisa Ravdin, PhD ^89^, Athena Lee, PhD ^89^, Ron Petersen ^23^, Thomas Neylan, MD ^1^, Jordan Grafman, PhD ^90^, Tom Montine, MD, PhD ^91^, Michael W. Weiner MD ^1^, Ronald Petersen ^3^, Devon Gessert , BS ^2^, Lindsey Hergesheimer, BS ^2^, Sarah Danowski, MA ^2^, Catherine Nguyen-Barrera, MS ^2^, Thomas Neylan, MD ^1^, Jacqueline Hayes ^1^, Shannon Finley ^1^, Danielle Harvey, PhD ^6^, Michael Donohue, PhD ^14^, Matthew Bernstein, PhD ^3^, Bret Borowski, RT ^23^, Matt Senjem, MS ^23^, Kejal Kantarci ^23^, Chad Ward ^23^, Stephanie Rossi Chen, BA ^22^, Robert A. Koeppe, PhD ^26^, Norm Foster, MD ^92^, Tatiana M. Foroud, PhD ^8^, Steven Potkin, MD UC ^93^, Li Shen, PhD ^8^, Kelley Faber, MS, CCRC ^8^, Sungeun Kim, PhD ^8^, Kwangsik Nho, PhD ^8^, Kristi Wilmes, MS, CCRP ^94^, Lon S. Schneider, MD ^2^, Bryan M. Spann, DO, PhD ^2^, Helen Vanderswag, RN ^14^, Adam Fleisher, MD ^14^, Ajay Sood, MD, PhD ^42^, Kimberly S. Blanchard, DNP, APRN, NP-C ^42^, Debra Fleischman, PhD – Past Investigator Konstantinos Arfanakis, PhD ^42^, Dr. Ranjan Duara MD ^43^, Dr. Daniel Varon MD ^43^, Maria T Greig HP ^43^, Bonnie Goldstein, MS, NP ^49^, Kimberly S. Martin, RN ^49^, Gaby Thai, MD ^95^, Aimee Pierce, MD ^95^, Christopher Reist, MD ^95^, Beatriz Yanez, RN ^95^, Elizabeth Sosa, PhD ^95^, Megan Witbracht, PhD ^95^, Carl Sadowsky, MD ^96^, Walter Martinez, MD ^96^, Teresa Villena, MD ^96^, Howard Rosen, MD ^1^, David Perry ^1^, Gad Marshall, MD ^64^, Edward Zamrini, MD ^65^, Sheeba Nadarajah, PhD, RN ^67^, Elaine R. Peskind, MD ^91^, Eric C. Petrie, MD, MS ^91^, Gail Li, MD, PhD ^91^, Jerome Yesavage, MD ^97^, Joy L. Taylor, PhD ^97^, Steven Chao, MD, PhD ^97^, Jaila Coleman, BA ^97^, Jessica D. White, BA ^97^, Barton Lane, MD ^97^, Allyson Rosen, PhD ^97^, Jared Tinklenberg, MD ^97^, Michael Lin, PhD ^89^, Gloria Chiang, MD ^89^, Scott Mackin, PhD ^1^, Rema Raman, PhD ^2^, Gustavo Jimenez-Maggiora, MBS ^2^, Devon Gessert, BS ^2^, Jennifer Salazar, MBS ^2^, Caileigh Zimmerman, MS ^2^, Sarah Walter, MSc ^2^, Olusegun Adegoke, MSc ^2^, Payam Mahboubi, MPH ^2^, Erin Drake, MA ^98^, Mike Donohue, PhD ^2^, Craig Nelson, MD ^1^, David Bickford, BA ^1^, Meryl Butters, PhD ^28^, Michelle Zmuda, MA ^28^, Bret Borowski, RT ^3^, Jeff Gunter, PhD ^3^, Matt Senjem, MS ^3^, Kejal Kantarci, MD ^3^, Chad Ward, BA ^3^, Denise Reyes, BS ^3^, Kelley M. Faber, MS, CCRC ^8^, Kelly N. Nudelman ^8^, Yiu Ho Au, BA ^1^, Kelly Scherer, BS ^1^, Daniel Catalinotto, BA ^1^, Samuel Stark, BA ^1^, Elise Ong, BA ^1^, Dariella Fernandez, BA ^1^, Michelle Zmuda, BS ^28^

^1^ University of California, San Francisco, San Francisco, U.S.A., ^2^ University of Southern California, Los Angeles, U.S.A., ^3^ Mayo Clinic, Rochester, Rochester, U.S.A., ^4^ University of California, Berkeley, Berkeley, U.S.A., ^5^ University of Pennsylvania, Philadelphia, U.S.A., ^6^ University of California, Davis, Davis, U.S.A., ^7^ BWH/HMS, Boston, U.S.A., ^8^ Indiana University, Bloomington, U.S.A., ^9^ Washington University St. Louis, St. Louis, U.S.A., ^10^ Alzheimer’s Association, Chicago, U.S.A., ^11^ National Institute of Mental Health, Rochville, U.S.A., ^12^ Rush University, Chicago, U.S.A., ^13^ NIA, Bethesda, U.S.A., ^14^ University of California, San Diego, San Diego, U.S.A., ^15^ Denali Therapeutics, South San Francisco, U.S.A., ^16^ NIH, Bethesda, U.S.A., ^17^ Massachusetts General Hospital, Boston, U.S.A., ^18^ Biogen, Cambridge, U.S.A., ^19^ University of Wisconsin, Madison, Madison, U.S.A., ^20^ Washington University, St. Louis, U.S.A., ^21^ Eli Lilly , Indianapolis, U.S.A., ^22^ NCIRE / The Vererans Health Research Institute, San Francisco, U.S.A., ^23^ Mayo Clinic, Scottsdale, U.S.A., ^24^ University College London, London, U.K., ^25^ University of Southern California School of Medicine, Los Angeles, U.S.A., ^26^ University of Michigan, Ann Arbor, U.S.A., ^27^ Banner Alzheimer’s Institute, Phoenix, U.S.A., ^28^ University of Pittsburgh, Pittsburgh, U.S.A., ^29^ Perelman School of Medicine, University of Pennsylvania, Philadelphia, U.S.A., ^30^ Indiana University School of Medicine, Indianapolis, U.S.A., ^31^ UPenn School of Medicine, Philadelphia, U.S.A., ^32^ NCRAD/Indiana University School of Medicine, Indianapolis, U.S.A., ^33^ Prevent Alzheimer’s Disease 2020, Rockville, U.S.A., ^34^ National Institute on Aging, Bethesda, U.S.A., ^35^ Oregon Health & Science University, Portland, U.S.A., ^36^ University of California – San Diego, San Diego, U.S.A., ^37^ Baylor College of Medicine, Houston, U.S.A., ^38^ Columbia University Medical Center, New York, U.S.A., ^39^ Washington University, St. Louis, St. Louis, U.S.A., ^40^ University of Alabama - Birmingham, Birmingham, U.S.A., ^41^ Mount Sinai School of Medicine, New York, U.S.A., ^42^ Rush University Medical Center, Chicago, U.S.A., ^43^ Wien Center, Miami Beach, U.S.A., ^44^ Johns Hopkins University, Baltimore, U.S.A., ^45^ University of South Florida: USF Health Byrd Alzheimer’s Institute, Tampa, U.S.A., ^46^ New York University, New York, U.S.A., ^47^ Duke University Medical Center, Durham, U.S.A., ^48^ University of Kentucky, Lexington, U.S.A., ^49^ University of Rochester Medical Center, New York, U.S.A., ^50^ University of California Irvine IMIND, Irvine, U.S.A., ^51^ University of Texas Southwestern Medical School, Dallas, U.S.A., ^52^ Emory University, Atlanta, U.S.A., ^53^ University of Kansas, Medical Center, Kansas, U.S.A., ^54^ University of California, Los Angeles, Los Angeles, U.S.A., ^55^ Mayo Clinic, Jacksonville, Jacksonville, U.S.A., ^56^ Yale University School of Medicine, New Haven, U.S.A., ^57^ McGill Univ., Montreal-Jewish General Hospital, Montréal, Canada, ^58^ Sunnybrook Health Sciences, Ontario, Toronto, Canada, ^59^ U.B.C. Clinic for AD & Related Disorders, Vancouver, Canada, ^60^ St. Joseph’s Health Care, Petaluma, U.S.A., ^61^ Northwestern University, Evanston, U.S.A., ^62^ Nathan Kline Institute, Orangeburg, U.S.A., ^63^ Georgetown University Medical Center, Washington, U.S.A., ^64^ Brigham and Women's Hospital, Boston, U.S.A., ^65^ Banner Sun Health Research Institute, Sun City, U.S.A., ^66^ Boston University, Boston, U.S.A., ^67^ Howard University, Washington, U.S.A., ^68^ Case Western Reserve University, Cleveland, U.S.A., ^69^ University of California, Davis – Sacramento, Sacramento, U.S.A., ^70^ Dent Neurologic Institute, Orchard Park, U.S.A., ^71^ Parkwood Institute, London, Canada, ^72^ University of Wisconsin, Madison, U.S.A., ^73^ Banner Alzheimer's Institute, Phoenix, U.S.A., ^74^ Ohio State University, Columbus, U.S.A., ^75^ Albany Medical College, Albany, U.S.A., ^76^ University of Iowa College of Medicine, Iowa City, U.S.A., ^77^ Wake Forest University Health Sciences, Winston Salem, U.S.A., ^78^ Rhode Island Hospital, Providence, U.S.A., ^79^ Cleveland Clinic Lou Ruvo Center for Brain Health, Las Vegas, U.S.A., ^80^ Roper St. Francis Healthcare, Charleston, U.S.A., ^81^ Houston Methodist Neurological Institute, Houston, U.S.A., ^82^ Barrow Neurological Institute, Phoenix, U.S.A., ^83^ Vanderbilt University Medical Center, Nashville, U.S.A., ^84^ Long Beach VA Neuropsychiatric Research Program, Long Beach, U.S.A., ^85^ Butler Hospital Memory and Aging Program, Providence, U.S.A., ^86^ Neurological Care of CNY, East Syracuse, U.S.A., ^87^ Hartford Hospital, Olin Neuropsychiatry Research Center, Hartford, U.S.A., ^88^ Dartmouth-Hitchcock Medical Center, Lebanon, U.S.A., ^89^ Cornell University, Ithaca, U.S.A., ^90^ Rehabilitation Institute of Chicago, Feinberg School of Medicine, Northwestern University, Chicago, U.S.A., ^91^ University of Washington, Seattle, U.S.A., ^92^ University of Utah, Salt Lake City, U.S.A., ^93^ UC Irvine, Irvine, U.S.A., ^94^ NCRAD, Indianapolis, U.S.A., ^95^ University of California, Irvine, Irvine, U.S.A., ^96^ Premiere Research Inst (Palm Beach Neurology), West Palm Beach, U.S.A., ^97^ Stanford University, Stanford, U.S.A., ^98^ BWM/HMS, Boston, U.S.A.,
